# Catheter-related bloodstream infections in hemato-oncology paediatric patients

**DOI:** 10.1186/2047-2994-4-S1-P205

**Published:** 2015-06-16

**Authors:** HIG Giamberardino, U Coelho, N Bevilaqua, P Rett

**Affiliations:** 1 Epidemiology and Infection Control Department, Hospital Pequeno Principe, Brazil; 2 Pequeno Principe College, Curitiba, Brazil

## Introduction

Hemato-Oncology paediatric patients are more vulnerable to the development healthcare associated infections (HAI), due to the condition of immunosuppression, repeated hospitalizations, and long-term use of central venous catheters.

## Objectives

The objective was to evaluate the clinical and epidemiological profile of catheter related bloodstream infection (CRBI) in children with haematological and oncological disorders, in patients hospitalized in paediatric haematology and oncology unit, at Hospital Pequeno Principe.

## Methods

Retrospective study from January 2008 to December 2012 was performed. Data from the Epidemiology and Infection Control Department and medical records, of hospitalized paediatric patients at Hemato-Oncology unit, consisting of 12 beds, were analysed. All hospitalized patients, in the period of the study and diagnosed with HAI were included. The diagnostic criteria of Centers for Disease Control and Prevention (CDC) were used.

## Results

In the period of five years, were admitted to the haematology-oncology unit 3107 patients, and diagnosed 44 episodes of HAI (1.77%) in 32 patients. (1.02%). The mean age of the patients was 5 years old, 47% female and 53% male. The mean hospital stay was 6.6 days. The most prevalent topographies of infection were: 56.81% (25) CRBI 13.63% (6) pneumonia; 20:45% (9) urinary tract Infection; 15% (4) others topography (mouth, vascular access, gastrointestinal tract). The main underlying diseases were: 37.5% (12/32) malignant hematologic disease; 59.37% (19 / 32) solid tumours. Of the 25 episodes of CRBI the etiologic agent was identified in 84%: 28% (7/25) *Coagulase Negative Staphylococcus* (CONS), 16% (4/25) *Enterococcus sp*, 12% (2) *Pseudomonas aeruginosa*; 8% (2/25) *Klebsiella oxytoca*, 4.5% (1/25) *Candida guillierrmondii*; 4.5% (1/25) *Candida albicans;* 12% (3/25) others. Catheters used were: 42.85% (6/14) peripherally inserted central catheter, 35.71% (6/14) totally implantable venous access port devices; 21.42%(3/14) caheter not specified.

## Conclusion

Children undergoing immunosuppressive treatments and catheters are at high risk for the development CRBI. The study of epidemiology is essential to establish preventive measures. In consensus with the literature, the most prevalent agent was CONS.

## Disclosure of interest

None declared.

